# Design of Disruptors of the Hsp90–Cdc37 Interface

**DOI:** 10.3390/molecules25020360

**Published:** 2020-01-15

**Authors:** Ilda D’Annessa, Naama Hurwitz, Valentina Pirota, Giovanni Luca Beretta, Stella Tinelli, Mark Woodford, Mauro Freccero, Mehdi Mollapour, Nadia Zaffaroni, Haim Wolfson, Giorgio Colombo

**Affiliations:** 1 SCITEC-CNR, via Mario Bianco 9, 20131 Milan, Italy; ilda.dannessa@uniroma2.it; 2 Blavatnik School of Computer Science, Tel Aviv University, Tel Aviv 6997801, Israel; namih99@gmail.com; 3 Department of Chemistry, University of Pavia, via Taramelli 12, 27100 Pavia, Italy; valentina.pirota01@ateneopv.it (V.P.); mauro.freccero@unipv.it (M.F.); 4 Molecular Pharmacology Unit, Department of Applied Research and Technological Development, Fondazione IRCCS Istituto Nazionale Tumori, Via Amadeo 42, 20133 Milan, Italy; giovanni.beretta@istitutotumori.mi.it (G.L.B.); stella.tinelli@istitutotumori.mi.it (S.T.); nadia.zaffaroni@istitutotumori.mi.it (N.Z.); 5 Department of Urology, SUNY Upstate Medical University Syracuse, Syracuse, NY 13210, USA; woodform@upstate.edu (M.W.); mollapom@upstate.edu (M.M.); 6 Department of Biochemistry and Molecular Biology, SUNY Upstate Medical University Syracuse, Syracuse, NY 13210, USA; 7 Upstate Cancer Center, SUNY Upstate Medical University Syracuse, Syracuse, NY 13210, USA; 8 College of Medicine, SUNY Upstate Medical University Syracuse, Syracuse, NY 13210, USA

**Keywords:** Hsp90, cdc37, protein–protein interaction, peptide design

## Abstract

The molecular chaperone Hsp90 is a ubiquitous ATPase-directed protein responsible for the activation and structural stabilization of a large clientele of proteins. As such, Hsp90 has emerged as a suitable candidate for the treatment of a diverse set of diseases, such as cancer and neurodegeneration. The inhibition of the chaperone through ATP-competitive inhibitors, however, was shown to lead to undesirable side effects. One strategy to alleviate this problem is the development of molecules that are able to disrupt specific protein–protein interactions, thus modulating the activity of Hsp90 only in the particular cellular pathway that needs to be targeted. Here, we exploit novel computational and theoretical approaches to design a set of peptides that are able to bind Hsp90 and compete for its interaction with the co-chaperone Cdc37, which is found to be responsible for the promotion of cancer cell proliferation. In spite of their capability to disrupt the Hsp90–Cdc37 interaction, no important cytotoxicity was observed in human cancer cells exposed to designed compounds. These findings imply the need for further optimization of the compounds, which may lead to new ways of interfering with the Hsp90 mechanisms that are important for tumour growth.

## 1. Introduction

Cellular homeostasis is a fine-tuned regulated condition, strictly dependent on the correct assembly and functionality of the proteome. All living organisms have developed a series of strategies to assist proteins in acquiring and maintaining a functional fold, to avoid the formation of toxic aggregates, or to protect them from the effect of external injuries like heat shock stresses. Heat Shock Protein 90 (Hsp90) is a paradigmatic example of a molecular machine that is able to oversee all of these mechanisms. Hsp90 forms a family of molecular chaperones that play a pivotal role in safeguarding proteome balance. Hsp90 was first discovered to protect proteins from unfolding following heat stress, but later it was found to be constitutively expressed and able to promote conformational changes late in the folding processes of proteins, named clients, which are necessary to allow them to achieve an active state [1,2]. Hsp90′s clientele is represented by a plethora of different proteins with diverse activities and three-dimensional arrangements. Because of this, cells have evolved a mechanism to help Hsp90 to select the correct client from this complex ensemble of possibilities. In this context, specifically in higher organisms, given co-chaperones are able to load specific clients on Hsp90, modulating its activity in different cell tissues or along particular pathways [3]. Hsp90 and its co-chaperone systems are particularly overexpressed in transformed cells associated to disease states, especially cancer and neurodegeneration [4,5,6].

Disruption of the functions of Hsp90–co-chaperone systems could provide novel opportunities for the development of pharmacological leads and chemical tools to investigate the roles of chaperone complexes in different pathways. Selective disruption of Hsp90 interaction with a definite co-chaperone can selectively inhibit the activation of a subpart of the Hsp90 clientele, avoiding the indiscriminate shutdown of the multiple proteins at the basis of the toxicity observed for ATP-competitive inhibitors (vide infra). This, however, requires a full description of the Hsp90/client/co-chaperone complex at atomistic resolution. To date, due to the complexity of the system, this strategy has not been significantly pursued.

From a structural and biochemical point of view, Hsp90 is active as a dimer and couples client maturation with ATP hydrolysis through a complex conformational cycle [7,8,9]. Each protomer is comprised of an N-terminal domain (NTD) hosting the ATPase site, a middle domain (MD), mainly dedicated to interactions with clients and co-chaperones, and a C-terminal domain (CTD) which is primarily involved in the dimerization process [10,11,12]. The first generation of Hsp90 inhibitors is represented by molecules which target the ATP site, competing to bind with nucleotides, and thus inhibiting ATP hydrolysis and, consequently, all clients’ maturation [13]. It must be noted here that Hsp90 belongs to the GHKL superfamily, together with DNA gyrase B, histidine kinase and MutL, with which it shares ATP-binding determinants. Consequently, ATP-competitive inhibitors can have different off-targets, which may cause toxicity due to their side effects. Moreover, N-terminal-directed inhibitors tend to induce a heat shock response by activating HSF1, eventually resulting in an overexpression of the Hsp70 pathway, which ends up favouring cancer cell survival [14]. For these reasons, alternative strategies leading to selective and more specific inhibition of Hsp90 are highly desirable [15,16,17,18]. One such strategy is represented by the targeted disruption of protein–protein interactions.

In this framework, the release in 2016 of the Cryo-EM structure of the Hsp90/Cdk4/Cdc37 complex can represent a major breakthrough, allowing—for the first time—the observation of the complete arrangement and interaction of Hsp90 with a client and its co-chaperone [19]. This is of crucial importance not only to understand the mechanism of chaperone–client recognition and Hsp90-induced client maturation, but also for drug development. The client in this complex is a Cyclin-dependent kinase (Cdk), an enzyme that regulates the progression of the cell cycle, as well as transcription, mRNA processing, and the differentiation of nerve cells; Cdk abnormal activation is directly related to cancer onset and progression. Hsp90 promotes the activation of Cdks with the help of the co-chaperone Cdc37 [20]. The latter pre-processes and selects kinases for their entry in the chaperone cycle based on their structural stabilities [3].

Because Cdc37 co-chaperones different Cdks, inhibition of the Hsp90–Cdc37 interaction can affect the maturation of such kinases. The complex can thus be considered to be a platform/template for the design of new molecules aimed at disrupting such relevant interactions, affecting specific cellular pathways.

Here, we set out to study, through computational and theoretical approaches, the principal interaction patterns that stabilize the interface between Hsp90 and Cdc37. We then used this information as the basis for the design of peptidomimetics which mimic Cdc37 recognition determinants. Peptide-based mimicry of relevant protein–protein interactions (PPIs) can be aptly considered as an innovative tool to target the intricacies of chaperone-mediated mechanisms.

In this paper, we have combined the analysis of long MD simulations of the Hsp90/Cdk4/Cdc37 complex with novel (computational) peptide design and optimization methods which have been applied to retrieve peptide sequences with a predicted high binding affinity to the Hsp90 target. The peptide sequences that scored favorably were designed and tested in Co-IP experiments to show their ability to disrupt Hsp90 complexes and impact on the maturation and cell levels of kinase clients.

## 2. Results and Discussion

**Figure 1:**
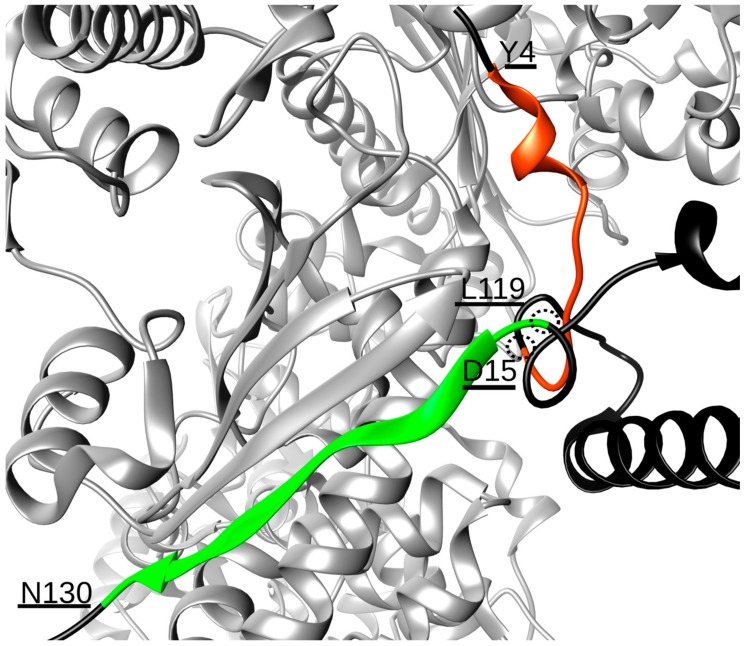
Interaction interface between protomer B of Hsp90 (light grey) and the N-terminal domain of Cdc37 (black). The two stretches of residues of Cdc37 responsible for the binding are highlighted in orange (residues Y4-D15) and green (L119–N130). The structural proximity of residues D15 and L119, which permits the two chains to join, is also underlined.

Extensive MD simulations of 12 μs of the Hsp90/Cdk4/Cdc37 complex revealed that Hsp90 and Cdc37 interact through a stable hydrogen bonding network established between two contact points, involving residues Y4-D15 and L119-N130 of Cdc37 (Table 1). Even if these two stretches of amino acids are far apart in sequence, they are proximal in the 3D folded structure of the co-chaperone in the complex, thus shaping a single interaction surface (Figure 1). The Y4-D15 sequence forms an unstructured motif, protruding into the Hsp90 NTDs interface, that contains residue S13, which is known to be a phosphorylation site crucial for regulating the interaction with the chaperone. Indeed, only when S13 is phosphorylatedcanCdc37 bind to Hsp90 to assist the loading of client kinases onto the chaperone [21,22]. Concerning the L119-N130 peptide, this sequence forms a β-strand that aligns with the Hsp90 β-strand formed by residues D314-E324 of protomer B, with which it establishes a number of hydrogen bonds (Table 1), thus becoming part of the Hsp90 five-stranded β-sheet (Figure 1). In terms of design, these data suggest the possibility to realize a sequence covering stretch Y4-D15, one covering L119-N130 and, given the spatial vicinity of the two, a single 25 residue-long sequence spanning both (Table 2). Ideally, such molecules should compete with Cdc37 for the Hsp90 surface, thus perturbing the correct assembly of the functional chaperone complex and impacting on client maturation.

To mimic the effects of S13 phosphorylation, while avoiding synthetic difficulties linked to the use of phosphorylated residues, glutamic or aspartic acid residues were used as substituents of S13. We first realized three different sequences, each 25 residues long, by joining D15 to L119 (Figure 1) and having a serine, an aspartic acid or a glutamic acid substitution (Table 2). The three peptides were labeled Cdc37p1, Cdc37p2 and Cdc37p3, respectively.

Next, we docked the three peptides on the structure of Hsp90 using the HADDOCK approach, suitable for protein–peptide docking [23]. Interestingly, the best solutions consistently show that all three peptides engage the surface of Hsp90 involved in Cdc37-binding with the same orientation, shown by the two stretches in the context of the entire protein, maintaining the network of hydrogen bonds detected by MD simulations (data not shown).

**Figure 2:**
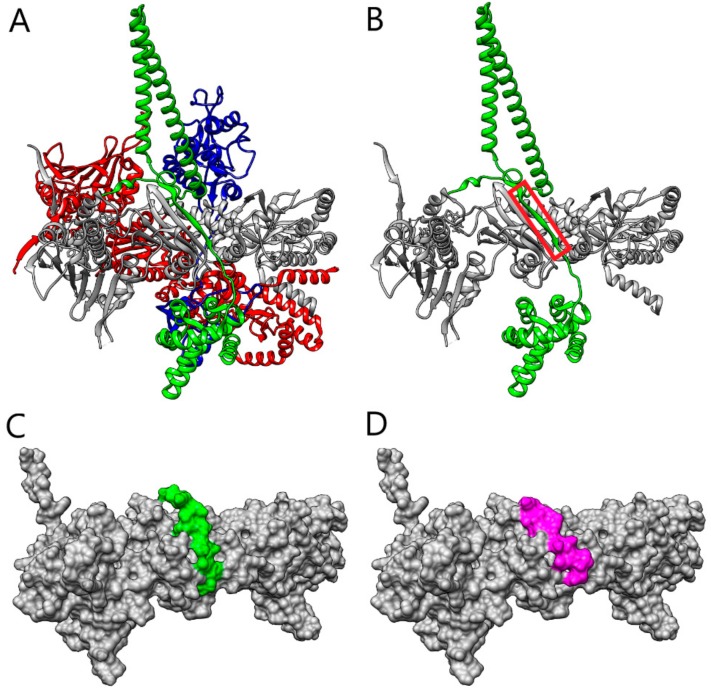
(**A**) The structure of Hsp90–Cdc37-Cdk4 complex (Hsp90 protomers A and B are in red and gray, respectively, Cdc37 is in green, and Cdk4 is in blue). (**B**) The structure of Hsp90B–Cdc37 (Hsp90B in grey, Cdc37 in green). The fragment of Cdc37 that was selected by the PepCrawler algorithm for the inhibitory peptide is marked in red. (**C**) The molecular surface of Hsp90B (grey), in the complex with the Cdc37p4 peptide retrieved by PepCrawler (green). (**D**) The molecular surface of Hsp90 (in grey), in complex with the Pep-Whisperer-designed peptide Cdc37p5 (magenta).

Parallel to this, we proceeded with the design of additional peptides based on the analysis of the Hsp90/Cdc37 interface (Figure 2A) through a bioinformatics approach. We sought to identify the region of Cdc37 that is crucial for the recognition of Hsp90 and which acts as an anchor fragment for triggering the interaction.

In the bioinformatics design, we first applied the PepCrawler algorithm [24] to excise—from the Cdc37 binding site with Hsp90B—a linear peptide with the highest binding affinity to Hsp90. The PepCrawler algorithm detects potential protein–protein interaction inhibitors by scanning the candidate binding interface to excise linear peptides which have predicted optimal binding to the target protein. In order to evaluate this binding energy, the algorithm efficiently generates alternative tightly-binding conformations of the excised peptides which allow for backbone and side-chain flexibility. Finally, conformations are sorted by evaluating the binding energy and steepness of the binding funnel. For peptides of length 10–15 it was found that a binding funnel with a slope above five is a good indicator for binding. Application of the PepCrawler algorithm resulted in the extraction of the peptide LSKDGFSKSMVN (labeled Cdc37p4), corresponding to the stretch of residues L119-N130, with a binding energy of −20.37 PepCrawler energy units (PEU) and a binding funnel with a steepness of 6.36. Figure 2B depicts the peptide fragment excised from the Cdc37 and Figure 2C depicts the modeled interaction of the peptide with Hsp90B. Next, we focused on improving the Cdc37p4 peptide by examining possible in silico mutations. The Pep-Whisperer algorithm (Hurwitz and Wolfson, in preparation) was applied to examine the mutations of subsets of the Cdc37p4 peptide amino acids in order to improve its PepCrawler binding energy and the steepness of the binding funnel. The Pep-Whisperer algorithm is guided by evolutionary information on the amino acids of the binding partner (Cdc37) in the interface by preferring more conserved residues. The best Pep-Whisperer-designed peptide was PSKDIFLKSMIN (labeled Cdc37p5), which includes four mutations of the original peptide and results in a binding energy of −47.28 PEU and a binding funnel with a steepness of 11.68.

Figure 2D depicts the modeled interaction of peptide Cdc37p5 with Hsp90B. Both Cdc37p4 and Cdc37p5 were subjected to further investigation by MD to test the stability of the interaction interface. The map of the hydrogen bond interactions between Hsp90 and the Cdc37p5-designed peptide, as detected along the MD simulation, is reported in Figure S1.

**Figure 3:**
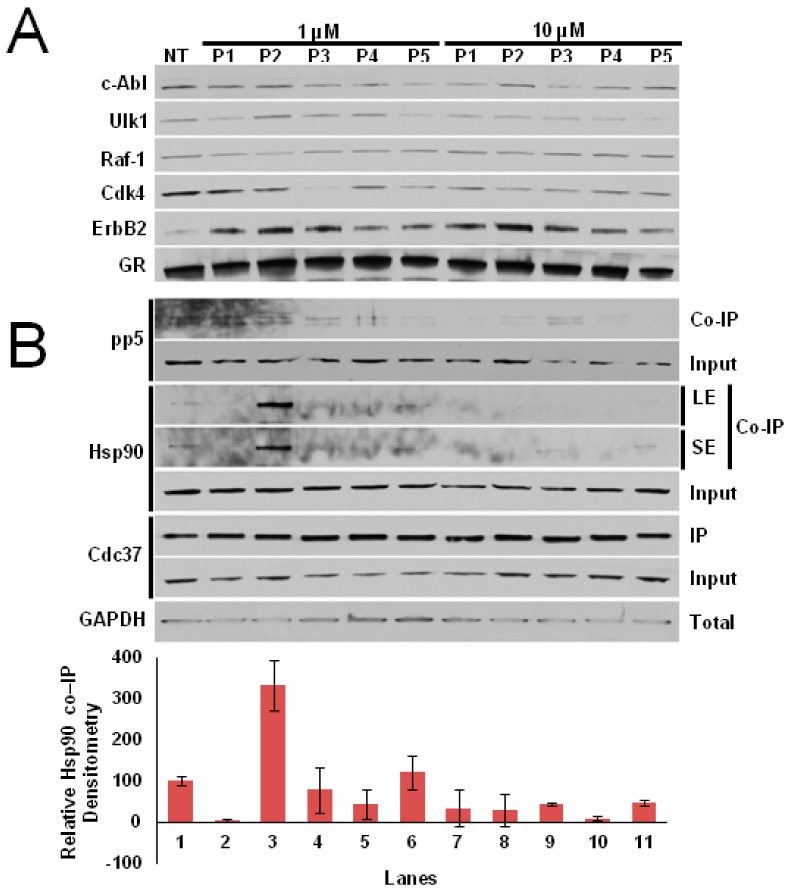
(**A**) Maturation of selected kinases detected by immunoblot assay in the presence of 1 µM (Lanes 2–6) or 10 µM (Lanes 7–11) of designed peptides. (**B**) Immunoprecipitation (IP) of Cdc37 in cell lysates in the presence of 1 µM (Lanes 2–6) or 10 µM (Lanes 7–11) of designed peptides. LE = long exposure, SE = short exposure.

The five peptides were then synthesized and tested in vitro for their ability to selectively inhibit Cdc37-dependent Cdks’ maturation, without affecting non-kinase clients. As depicted in (Figure 3A), Cdc37p3 and Cdc37p5 are able to inhibit Cdk4 maturation at 1 μM concentration (Lanes 4 and 6), while—at a concentration of 10 μM—each peptide is able to decrease the amount of mature client protein (Lanes 7–11). On the other hand, no effect on client protein level following treatment with the peptides can be detected for non-Cdc37-dependent kinases. To confirm that the decrease in Cdk4 levels in the presence of the peptides was due to a direct effect on the Hsp90/Cdc37 machinery, we performed an immunoprecipitation (IP). Clearly, the peptides are able to trap Hsp90 at 1 μM concentration, with the exception of Cdc37p2, which becomes active at 10 μM (Figure 3B), thus abrogating its interaction with Cdc37.

Interestingly, the Cdc37-derived peptides are also able to trap PP5, identified as the phosphatase dedicated to the specific dephosphorylation of Cdc37-S13, which is necessary to activate Hsp90 kinase clients [25,26]. 

Overall, the peptides significantly perturb, at the molecular level, interactions that may be important for cell survival in disease development—for instance, in cancer and/or neurodegeneration. While expectedly not potent, given their nature, we suggest that the effects of the peptides on the Hsp90 complex may synergize with those determined by small-molecule drugs, opening up novel therapeutic opportunities in exploiting Hsp90 networks to target cancer.

The results obtained with our computationally designed Cdc37-derived peptides are thus interesting in setting the stage for their further modification, aimed at developing more active molecules able to selectively disrupt Hsp90–Cdc37 interaction. First, based on MD analyses, we are designing additional mutations, namely non-natural amino acids or staples, to be introduced into the sequences in order to stabilize the active conformation of the peptides/peptidomimetics and optimize their membrane permeability, proteolytic stability and drug-like potential.

## 3. Materials and Methods

### 3.1. Computational Protocol

The three-dimensional coordinates of the Hsp90/Cdc37/Cdk4 complex were extracted from the structure deposited in the Protein Data Bank (PDB) with PDB code 5FWK [19]. The simulative system was built by introducing the complex in a triclinic box filled with 101,532 TIP3P [27] water molecules and was rendered electroneutral by the addition of 70 atoms of sodium counterions. The final system, in the presence of two ATP molecules, each one bound to one Hsp90 subunit, consisted of 331,875 atoms. The topology of the system was obtained with the Amber14 force field using the Amber14 suite [28].

The system was first minimized with around 10,000 steps of steepest descent followed by 10,000 steps of conjugate gradient, which was then equilibrated by running 1 ns of simulation with a 1 fs timestep via a temperature increase from 50 to 250 K with intervals of 50 K. After that, 1 μs of molecular dynamics simulation was carried out with a 2 fs timestep using Amber14 pmemd.cuda [28] in periodic boundary conditions, with a cut-off of 8 Å for the evaluation of short-range non-bonded interactions, and the Particle Mesh Ewald method for long-range electrostatic interactions [29]. The temperature was kept constant at 300 K with Langevin dynamics [30], whereas pressure was fixed at 1 Atmosphere through the Langevin piston method [31]. The bond lengths of solute and water molecules were restrained with the SHAKE [32] and SETTLE [33] algorithms, respectively. Finally, the simulation trajectory was collected for analysis with the Gromacs 4.6 [34] package, or with code written in-house. 

Based on the evaluation of the hydrogen bonding network established by Hsp90 and Cdc37 along this trajectory, we have selected the residues of Hsp90 needed to drive the docking of the Cdc37-derived peptide with Hsp90, using the Haddock webserver [35]. Thus, the Hsp90 residues interacting with Cdc37, as reported in Table 1, were used as active residues (AIRs) in the docking of Cdc37p1, Cdc37p2 and Cdc37p3. The docking poses obtained are classified based on the Haddock score, which is a sum of different contributions for the binding. The lowest is the Haddock score; the highest is the affinity between ligand and receptor [36]. In the case of the three peptides, the Haddock score for the best poses are 152.1 +/− 3.0, −148.5 +/− 3.0 and −146.6 +/− 10.0, respectively.

PepCrawler (http://bioinfo3d.cs.tau.ac.il/PepCrawler/) was applied in the protein–protein mode with PDB 5FWK as the input, where chain B (representing HSP90B) was defined as “receptor” and chain E (Cdc37) as “ligand”. A full RRT run was executed with the default energy parameters. The program automatically extracted peptide LSKDGFSKSMVN (subsequently labeled Cdc37p4), corresponding to the stretch of residues L119-N130, as the lowest energy (−20.37 PEU) and steepest funnel (6.36) exhibiting a consecutive linear peptide in the interface. This peptide served as the input to the newly developed PepWhisperer program (Hurwitz and Wolfson, in preparation) which, after exploring evolutionary favorable point mutations, returned—as its top output—the peptide PSKDIFLKSMIN (subsequently labeled Cdc37p5), which included four mutations compared to the input peptide and resulted in an energy of −47.28 PEU and a binding funnel with a steepness of 11.68.

### 3.2. Peptide Synthesis

Protected amino acids, rink amide resin and other reagents for peptide synthesis and solvents were purchased from Sigma-Aldrich.

Peptide purifications were performed on an Agilent Technologies 1260 Infinity preparative HPLC equipped with a diode array detector, using a Waters XSelectHSS C18 colum (2.5 μm, 50 × 4.6 mm). LC-MS/MS data were recorded using a LCQ ADV MAX ion-trap mass spectrometer, with an ESI ion source. The system was run in automated LC-MS/MS mode and used a surveyor HPLC system (Thermo Finnigan, San Jose, CA, USA) equipped with a BEH Acquity UPLC column (1.7 µm) 2.1 × 50 mm. MS/MS experiments by collision-induced dissociation were performed with an isolation width of 2 Th (*m*/*z*); the activation amplitude was around 35% of the ejection radiofrequency amplitude of the instrument.

The peptides were synthesized using the standard Fmoc solid-phase synthesis on a semi-automatic synthesizer (Biotage® Initiator + SP Wave; Uppsala, Sweden). Rink-amide resin (loading 0.37 mmol/g) was used as a solid support, which yielded the peptides that were amidated at the C-terminus.

Fmoc Rink Amide resin was pre-swelled for 3 h in DMF and was submitted to the preliminary Fmoc-deprotection step, using 20% (*v*/*v*) piperidine in DMF. The amino acid coupling reactions were performed using 5 mol equiv. versus resin sites of a.a. in the presence of five equiv. of ethyl(hydroxyimino)cyanoacetate (Oxyma Pure), as auxiliary nucleophile, and five equiv. of *N*,*N*′-diisopropylcarbodiimide (DIC) as a coupling agent in 1:1 NMP and DCM mixture. The mixtures were heated at 75 °C using microwaves and left to react for 15 min, except for H residue, for which a room temperature coupling of 1 h was conducted.

The requirement of a recoupling step was assessed using a Kaiser test kit.

Deprotection of the Fmoc group was performed by treating the resin twice with 20% piperidine in DMF (3 and 10 min, r.t.). To reduce the levels of aspartimide-related impurities, Oxyma Pure (0.1 M) was added to the Fmoc removal solution after the coupling of the first D residue.

At the end of the synthesis, to simultaneously detach the peptide from the resin support and remove all the side-chain protecting groups of the amino acids, the dry resin was placed in a flask and a solution of 94% trifluoroacetic acid (TFA, 25 mL for 1 g of resin), 1,2-ethanedithiol (EDT, 2.5%) triisopropyl silane (1%), and water (2.5%) was added. EDT is necessary to suppress the acid-catalysed M oxidation.

After stirring for 3 h, the resin was removed by filtration under reduced pressure and cold diethyl ether was added to filtrates in order to induce the precipitation of the peptides.

The mixture was cooled and left in the fridge overnight to further assist the precipitation. The crude peptides were filtered and washed with cold diethyl ether then were purified by preparative HPLC, using 0.1% TFA in water and CH3CN as eluents. The following method was used (method A): flow: 30 mL/min; gradient: isocratic flow over 2 min 80% of aqueous solvent, reducing gradually to 70% aqueous solvent over 14 min, then isocratic flow for 4 min (λ detection: 210, 254 and 280 nm). The products were then lyophilized, yielding white solids, which were characterized by Electrospray Ionisation Mass Spectrometry (ESI-MS) (See SI, Table 3). The following method was used (method B): flow: 0.3 mL/min; gradient: isocratic flow over 2 min 95% of aqueous solvent, changing gradually to 60% aqueous solvent over 18 min (λ detection: 210, 254 and 280 nm). Synthetic sequences were next used for cell tests [37].

### 3.3. Peptide Treatment

Cultured HEK293 cells were seeded 24 h prior to treatment. Peptides were added to cells at 50% confluency at the indicated concentrations and incubated for 24 h, followed by protein extraction as described below.

### 3.4. Protein Extraction, Immunoprecipitation and Immunoblotting

Protein extraction from HEK293 cells was carried out using methods previously described [Woodford, Cell Reports (2016)]. For immunoprecipitation, protein lysates were incubated with a Cdc37 antibody for 2 h, followed by incubation with protein G agarose (Qiagen) for 2 h at 4 °C. Immunopellets were washed four times with fresh lysis buffer (20 mM Tris (pH 7.4), 100 mM NaCl, 1 mM MgCl_2_, 0.1% NP40, protease inhibitor cocktail (Roche), and PhosSTOP (Roche)) and eluted with 5× Laemmli buffer. Precipitated proteins were separated by SDS-PAGE and transferred to nitrocellulose membranes. Co-immunoprecipitated proteins were detected by immunoblotting (Thermo Scientific Pierce ECL2) with the indicated antibodies (Ulk1, GR, c-Abl, Raf-1, pp5—Cell Signaling Technology. ErbB2, Cd4—Santa Cruz Biotechnology. Hsp90, GAPDH—Enzo Life Sciences. Cdc37—StressMarq), diluted in 5% non-fat dry milk reconstituted in TBST.

## Figures and Tables

**Table 1:** Percentage of the existence of the hydrogen bonds occurring between Hsp90 and Cdc37 along the MD simulation of the Hsp90/Cdk4/Cdc37 complex.

Hsp90:Cdc37 Hydrogen Bonds	
VAL318:MET128	99.76
HIS320:LYS126	90.79
SER322:PHE124	88.28
GLU324:LYS121	86.99
GLN326:LEU118	73.62
LEU327:SER119	47.11
LYS399:ASP8	51.90
LYS402:ASP14	66.62
LYS406:pSER13	78.92
THR446:pSER13	49.77
ASN389:TYR4	68.58
LEU327:SER120	63.79
GLU414:SER127	92.02
LEU316:ASN130	92.05
HIS315:ASN130	72.82

**Table 2:** Amino acids’ composition of the sequences designed, synthesized and tested.

Peptides Sequence	Peptide Length	Code	Purity
NYSVWDHIEVSDDLSKDGFSKSMVN	25	Cdc37p1	>95%
NYSVWDHIEVDDDLSKDGFSKSMVN	25	Cdc37p2	>95%
NYSVWDHIEVEDDLSKDGFSKSMVN	25	Cdc37p3	>95%
LSKDGFSKSMVN	12	Cdc37p4	>95%
PSKDIFLKSMIN	12	Cdc37p5	>95%

**Table 3:** Characterization data of peptides synthetized, obtained by ESI-MS in positive-ion method.

Peptides Code	Mol. Wt.	ESI-MS Data (*m*/*z*)
Cdc37p1	2872.08	1436.6 (p1-2H^2+^); 958.2 (p1-3H^3+^); 719.1 (p1-4H^4+^)
Cdc37p2	2900.09	1450.8 (p2-2H^2+^); 967.6 (p2-3H^3+^); 726.1 (p2-4H^4+^)
Cdc37p3	2913.13	1457.6 (p3-2H^2+^); 972.0 (p3-3H^3+^); 729.7 (p3-4H^4+^)
Cdc37p4	1311.51	1311.6 (p4-1H^+^); 656.5 (p4-2H^2+^); 438.1 (p4-3H^3+^)
Cdc37p5	1391.68	1391.7 (p5-1H^+^); 696.5 (p5-2H^2+^); 464.7 (p5-3H^3+^)

## Supplementary Materials

The following are available online at https://www.mdpi.com/1420-3049/25/2/360/s1, Peptides characterization by UPLC and ESI-MS; Figure S1: Cdc37p5-Hsp90 Hydrogen bonds network.
