# Supplementary material for: Design of Disruptors of the Hsp90–Cdc37 Interface
Source: Molecules. 2020 Jan 15;25(2):360. doi: 10.3390/molecules25020360 (PMC7024268; doi:10.3390/molecules25020360)
Supplement: Supplementary file 1 [file molecules-25-00360-s001.pdf]

Design of disruptors of the Hsp90-Cdc37 interface.

Ilda D'Annessa, Naama Hurwitz, Valentina Pirota, Giovanni Luca Beretta, Stella Tinelli, Mark Woodford, Mauro Freccero, Mehdi Mollapour, Nadia Zaffaroni, Haim Wolfson,* Giorgio Colombo*

UPLC characterization (λ = 210 nm, method B)

Cdc37p1:

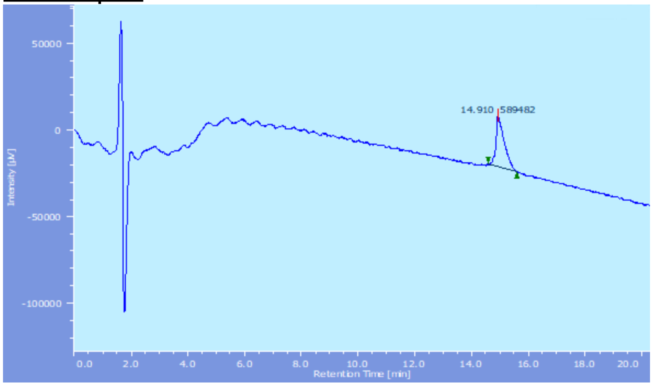

Cdc37p2:

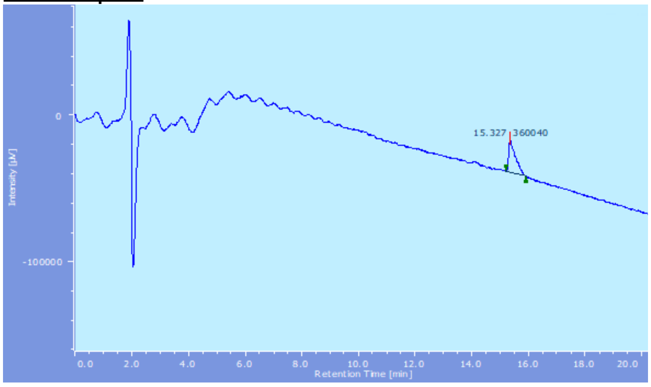

Cdc37p3:

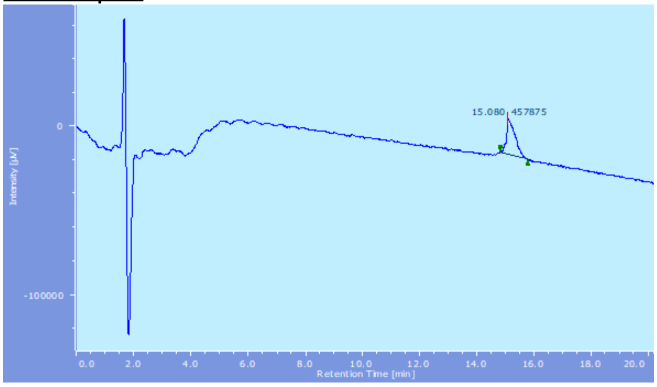

Cdc37p4:

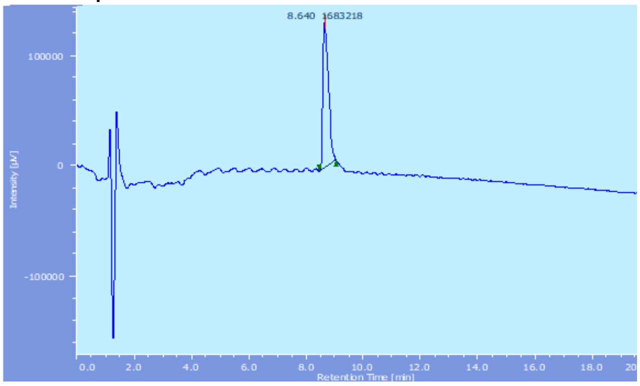

Cdc37p5:

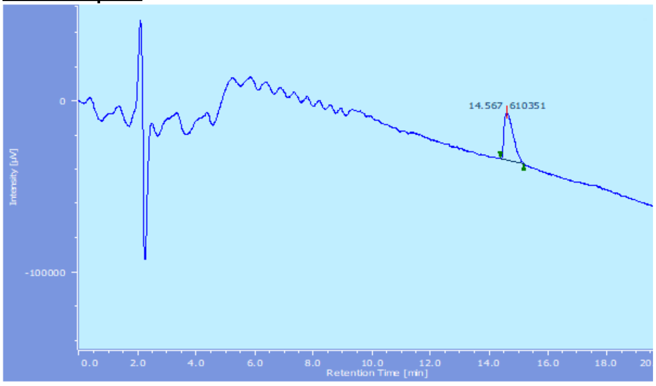

ESI-MS characterization

Cdc37p1

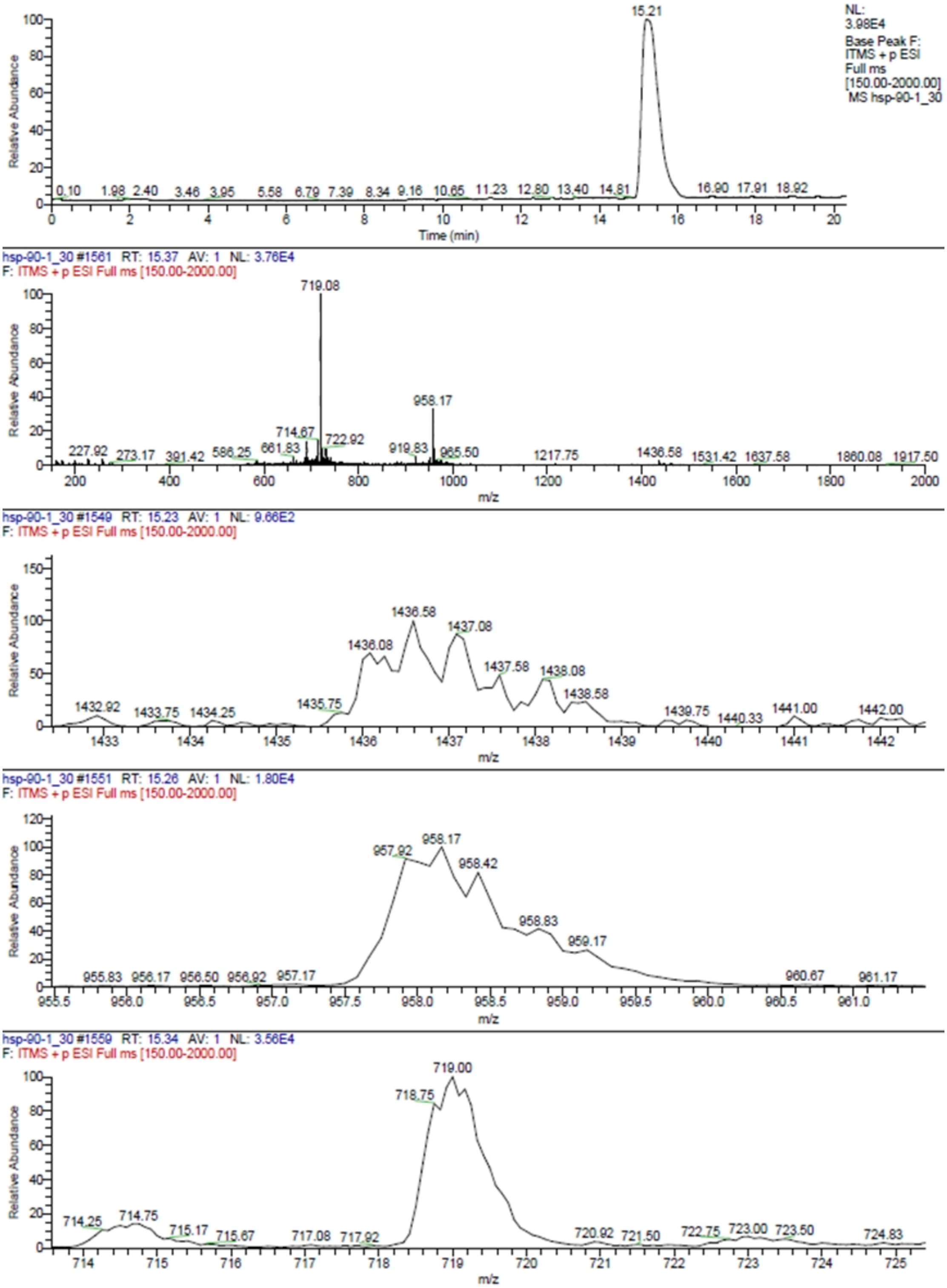

Cdc37p2

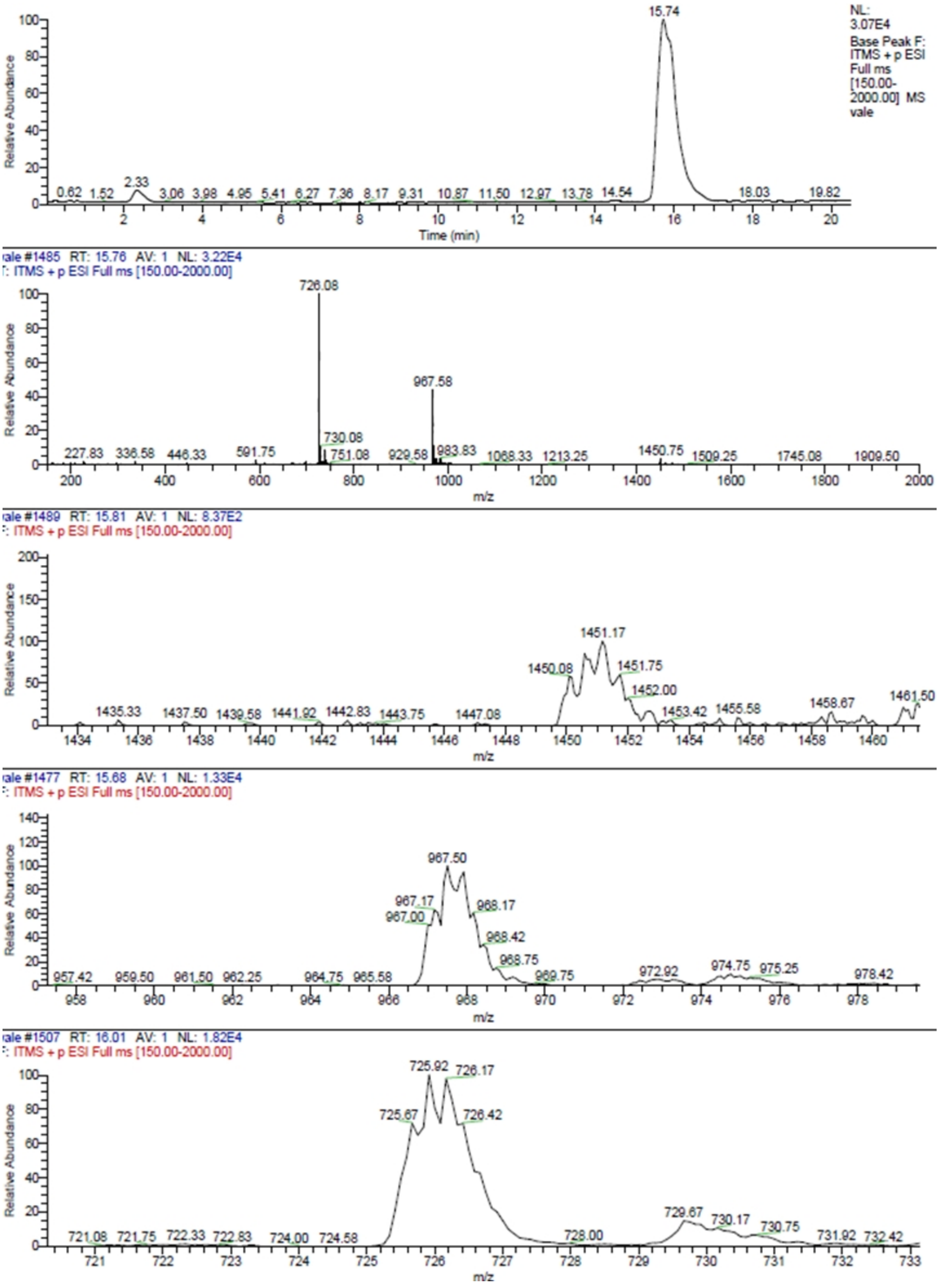
NL: 3.07E4 Base Peak F: ITMS + p ESI Full ms [150.00-2000.00] MS vale
15.74
Relative Abundance
Time (min)
vale #1485 RT: 15.76 AV: 1 NL: 3.22E4
T: ITMS + p ESI Full ms [150.00-2000.00]
726.08
967.58
m/z
vale #1489 RT: 15.81 AV: 1 NL: 8.37E2
T: ITMS + p ESI Full ms [150.00-2000.00]
1451.17
1450.08
1451.75
1452.00
vale #1477 RT: 15.68 AV: 1 NL: 1.33E4
T: ITMS + p ESI Full ms [150.00-2000.00]
967.50
967.17
967.00
968.17
968.42
968.75
vale #1507 RT: 16.01 AV: 1 NL: 1.82E4
T: ITMS + p ESI Full ms [150.00-2000.00]
725.92
726.17
725.67
726.42
729.67
730.17
730.75

Cdc37p3

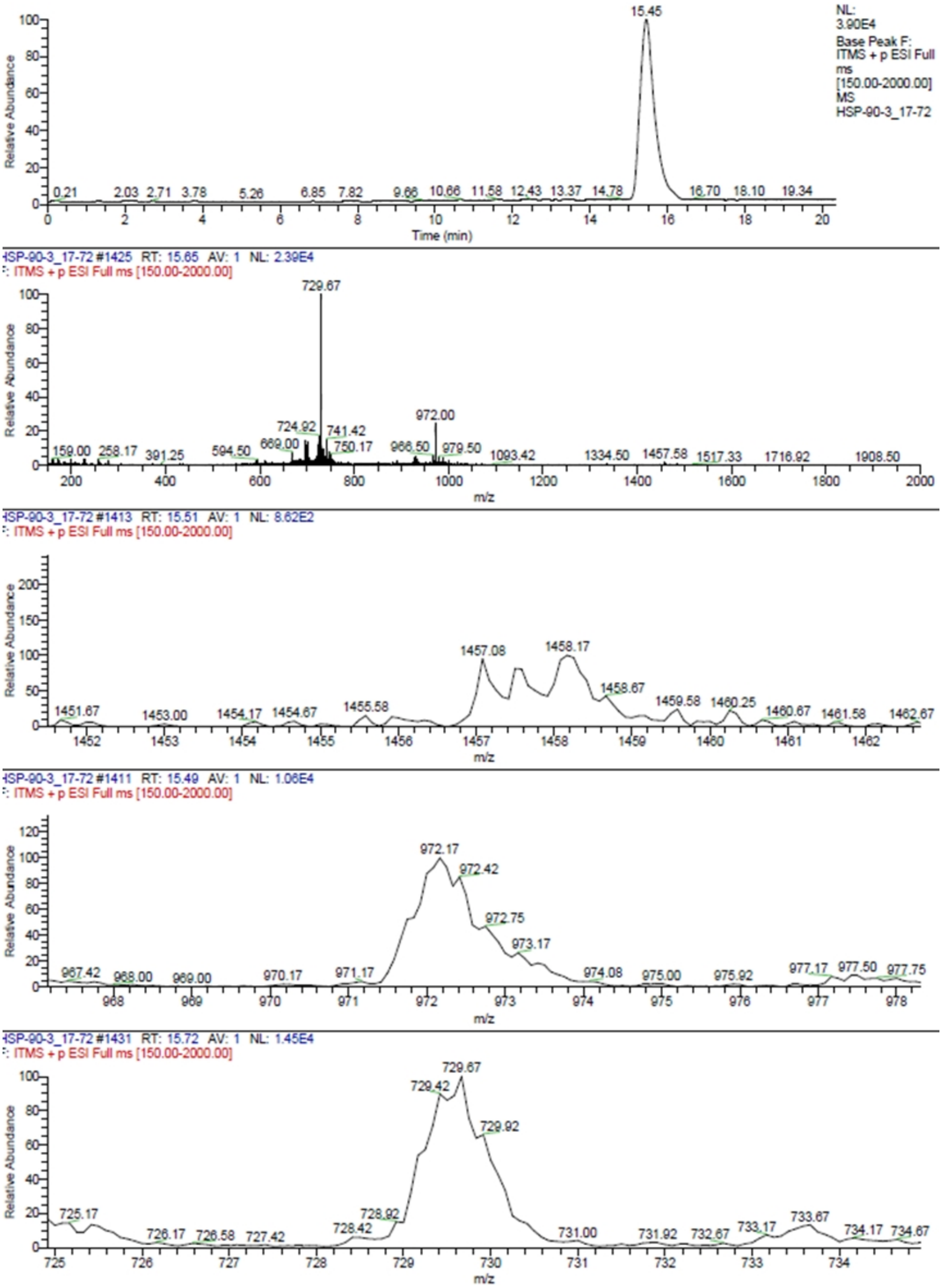

NL:
3.90E4
Base Peak F: ITMS + p ESI Full ms [150.00-2000.00] MS HSP-90-3_17-72
Relative Abundance
Time (min)
HSP-90-3_17-72 #1425 RT: 15.65 AV: 1 NL: 2.39E4
F: ITMS + p ESI Full ms [150.00-2000.00]
m/z
HSP-90-3_17-72 #1413 RT: 15.51 AV: 1 NL: 8.62E2
F: ITMS + p ESI Full ms [150.00-2000.00]
HSP-90-3_17-72 #1411 RT: 15.49 AV: 1 NL: 1.06E4
F: ITMS + p ESI Full ms [150.00-2000.00]
HSP-90-3_17-72 #1431 RT: 15.72 AV: 1 NL: 1.45E4
F: ITMS + p ESI Full ms [150.00-2000.00]

Cdc37p4

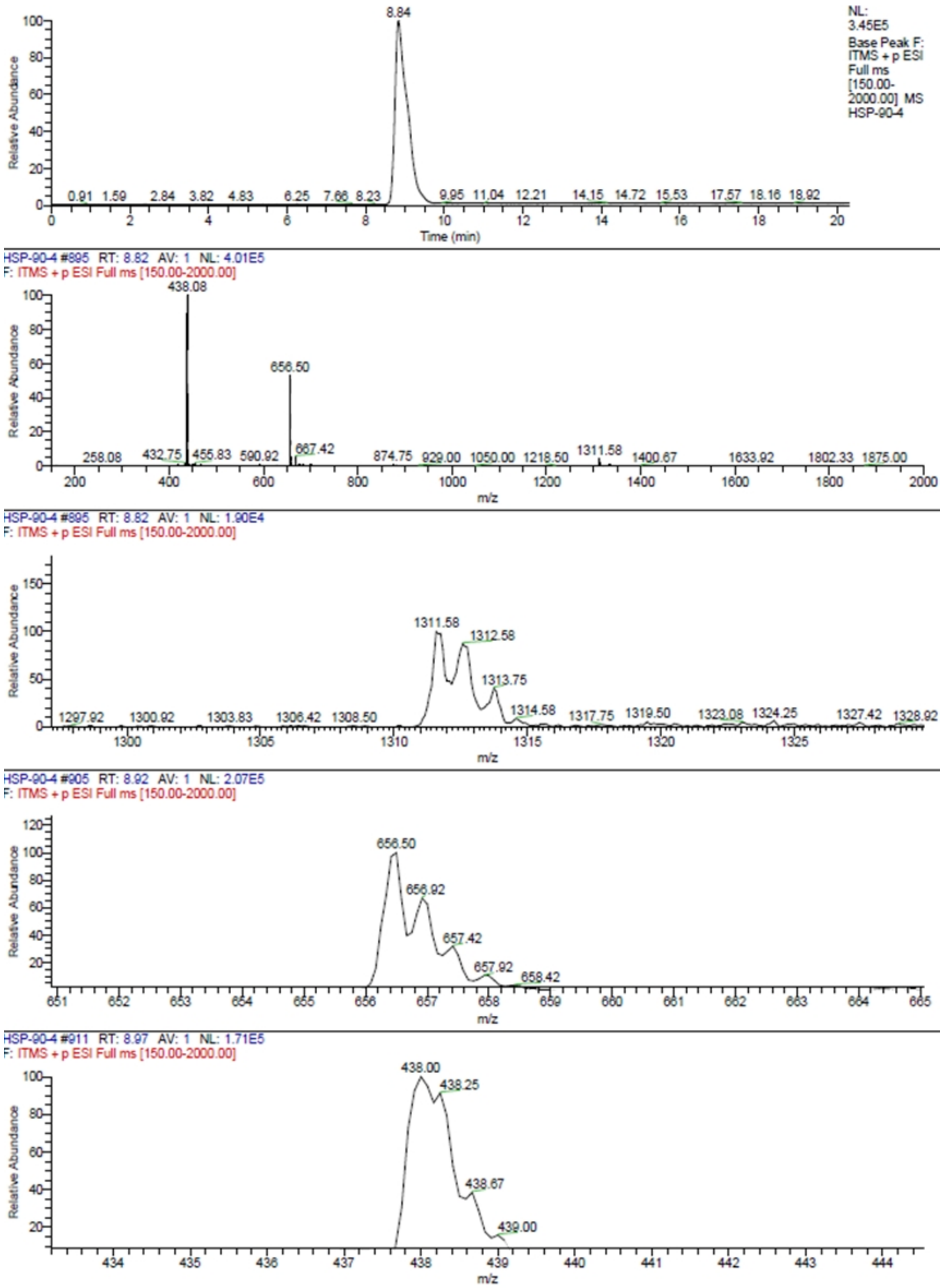
NL: 3.45E5
Base Peak F: ITMS + p ESI Full ms [150.00-2000.00] MS HSP-90-4
8.84
Relative Abundance
Time (min)
HSP-90-4 #895 RT: 8.82 AV: 1 NL: 4.01E5
F: ITMS + p ESI Full ms [150.00-2000.00]
438.08
656.50
m/z
HSP-90-4 #895 RT: 8.82 AV: 1 NL: 1.90E4
F: ITMS + p ESI Full ms [150.00-2000.00]
1311.58
1312.58
1313.75
HSP-90-4 #905 RT: 8.92 AV: 1 NL: 2.07E5
F: ITMS + p ESI Full ms [150.00-2000.00]
656.50
656.92
657.42
657.92
658.42
HSP-90-4 #911 RT: 8.97 AV: 1 NL: 1.71E5
F: ITMS + p ESI Full ms [150.00-2000.00]
438.00
438.25
438.67
439.00

Cdc37p5

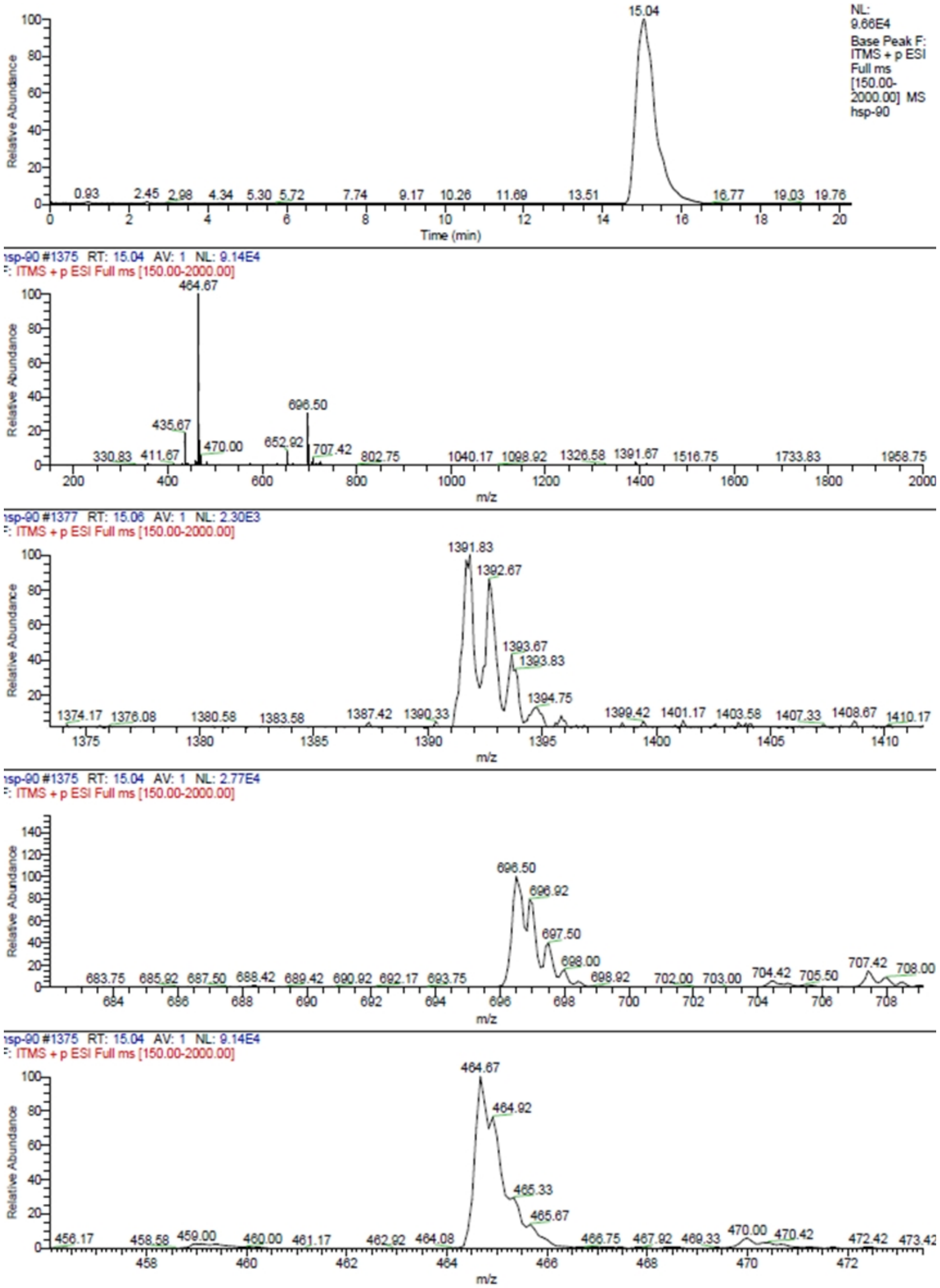

15.04
NL: 9.66E4
Base Peak F: ITMS + p ESI Full ms [150.00-2000.00] MS hsp-90
Relative Abundance
Time (min)
hsp-90 #1375 RT: 15.04 AV: 1 NL: 9.14E4
F: ITMS + p ESI Full ms [150.00-2000.00]
464.67
696.50
435.67
470.00
652.92
707.42
m/z
hsp-90 #1377 RT: 15.06 AV: 1 NL: 2.30E3
F: ITMS + p ESI Full ms [150.00-2000.00]
1391.83
1392.67
1393.67
1393.83
1394.75
hsp-90 #1375 RT: 15.04 AV: 1 NL: 2.77E4
F: ITMS + p ESI Full ms [150.00-2000.00]
696.50
696.92
697.50
698.00
hsp-90 #1375 RT: 15.04 AV: 1 NL: 9.14E4
F: ITMS + p ESI Full ms [150.00-2000.00]
464.67
464.92
465.33
465.67

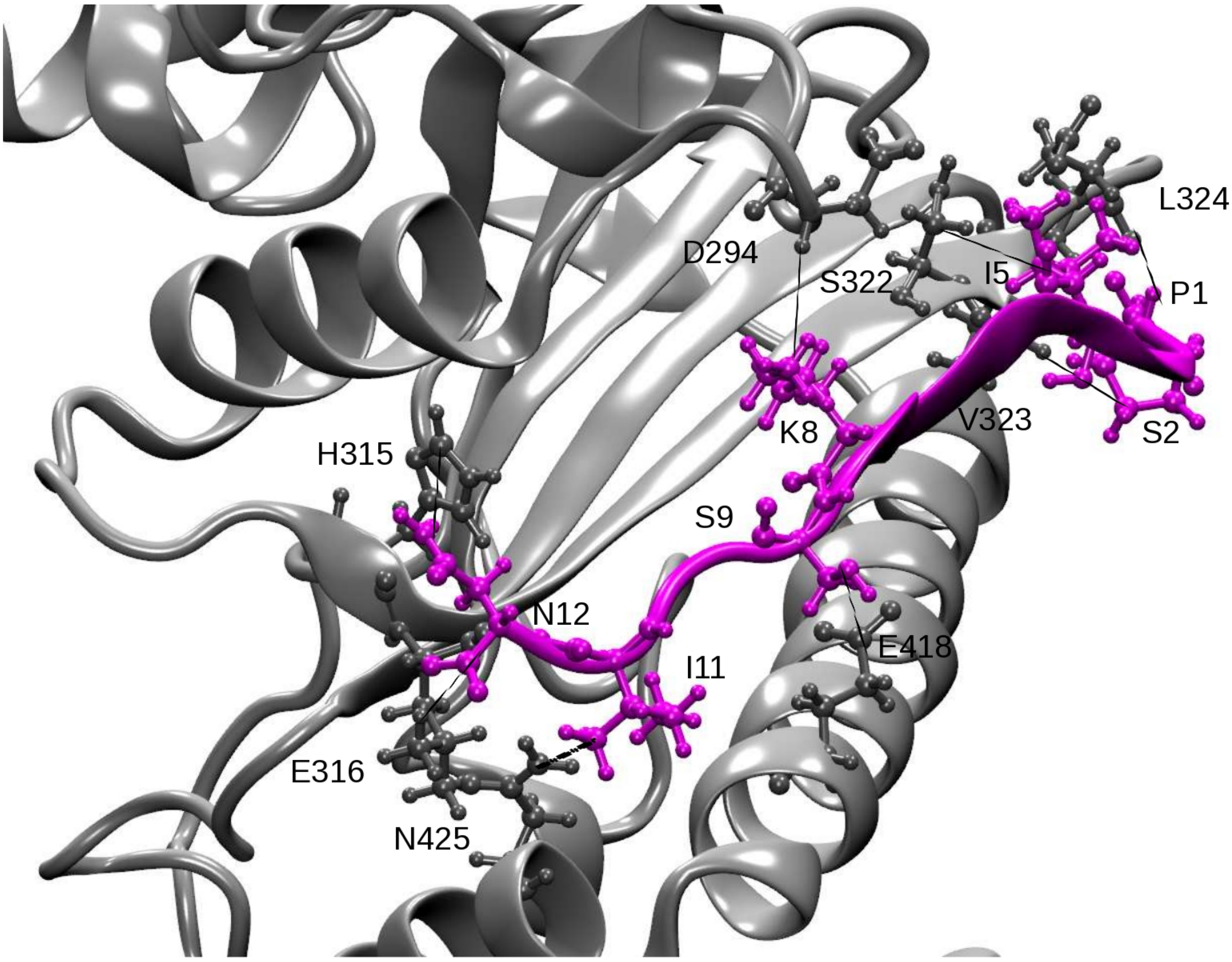

Figure S1. Map of the hydrogen bonds network between Hsp90 (light grey) and the newly designed peptide Cdc37p5 (magenta) as detected along molecular dynamics simulation of the Hsp90-Cdc37p5 complex.
